# Psychosocial Aspects of Cystic Fibrosis: A Mixed-Methods Systematic Review

**DOI:** 10.3390/healthcare14030351

**Published:** 2026-01-30

**Authors:** Maria Inês Griff, Rita Santos, Carmen Trumello, Tânia Brandão

**Affiliations:** 1 William James Center for Research, Ispa—Instituto Universitário, Rua Jardim do Tabaco, 44, 1149-041 Lisboa, Portugal; 27232@alunos.ispa.pt; 2 School of Psychology, Ispa—Instituto Universitário, Rua Jardim do Tabaco, 44, 1149-041 Lisboa, Portugal; 31538@alunos.ispa.pt; 3 Department of Psychology, University “G. d’Annunzio”, Via dei Vestini, 66100 Chieti, Italy; c.trumello@unich.it

**Keywords:** cystic fibrosis, psychosocial adaptation, mixed-methods systematic review

## Abstract

**Highlights:**

**What are the main findings?**

- Psychosocial aspects (e.g., coping, social support) were identified as central to the CF patient experience, shifting the focus beyond biological survival.
- Advances in treatment have significantly increased life expectancy, introducing new psychosocial challenges for adults with CF (e.g., management of visible symptoms, stigma in the workplace, financial instability).

**What are the implications of the main findings?**

- There is a need for integrated care models that address both clinical symptoms and psychosocial factors, such as reproductive goals and financial literacy, to support CF patients as they transition into adulthood.
- Digital and group psychological interventions are important to foster universality and boost peer connection, while maintaining clinical safety to prevent cross-contamination between CF patients.

**Abstract:**

**Background/Objectives**: Cystic fibrosis (CF) is a genetic condition with an increasing life expectancy in recent years. As a result, addressing psychosocial aspects in this population has become an increasingly important concern. This mixed-methods systematic review aimed to update the current knowledge on the psychosocial aspects of living with CF in adults. **Methods**: Following PRISMA guidelines, a literature search was conducted in November 2024 across several databases, including Scopus, ScienceDirect, Academic Search Complete, MEDLINE, Supplemental Index, Complementary Index, APA PsycInfo, Business Source Complete, SciELO, and the Directory of Open Access Journals via EBSCO. **Results**: Of the 701 articles retrieved, 24 were analyzed, including a total of 2023 participants (mean age: 31.2 years; 57.2% female). Quantitative findings identified optimistic coping as the most frequent strategy associated with improved survival. High social support and gratitude emerged as key factors for treatment adherence and quality of life, while depression remained the primary mental health concern. Qualitatively, the findings highlighted concerns with adult life transitions and financial stressors. Participants described experiences of social stigma and embarrassment linked to chronic symptoms, often leading to selective disclosure to avoid discrimination. **Conclusions**: This review confirms that psychosocial factors are central to the adult CF experience, shifting the focus beyond biological survival and highlighting areas that require clinical intervention. As life expectancy increases, clinical care must evolve to incorporate interventions that address these factors to improve mental health and overall quality of life (QoL), ensuring that patients are supported through the unique challenges of extended adulthood.

## 1. Introduction

Cystic fibrosis (CF) is a genetic, autosomal recessive disorder characterized by reduced or absent CFTR protein function [1]. This protein deficiency results in the accumulation of mucus in various organs, including the lungs, liver, pancreas, and gastrointestinal tract [1]. Consequently, the most prominent clinical manifestations of CF include chronic pulmonary infections, leading to bronchiectasis and progressive lung failure, chronic hepatobiliary disease, exocrine pancreatic insufficiency, intestinal obstruction, and male infertility [2]. As the disease progresses, individuals may also experience shortness of breath, exercise intolerance, chronic sinusitis, nasal polyps [3], and the potential development of diabetes [4]. CF primarily affects the Caucasian population, with a global prevalence of approximately 80,000 individuals [5]. However, between 2008 and 2021, only about 50,000 patients were registered in the European Cystic Fibrosis Society Database [6].

Contrary to what occurred when CF was first described in 1938, individuals with CF have seen their life expectancy increase significantly due to the progressive understanding of the disease and, consequently, the development of more effective treatments [2]. These treatments include the use of antibiotics to manage pulmonary infections, dietary modifications aimed at increasing caloric and fat intake, the use of pancreatic enzymes, anti-pseudomonas therapies, mucolytics, prenatal screening for early identification and intervention, and, most recently, treatment with CFTR modulators [7]. Additionally, lung transplantation has emerged as an option for extending life in patients who are considered suitable candidates [8]. However, complications from lung transplants have become the second leading cause of death in patients with CF [9]. As a consequence of these treatments, the financial costs associated with this disease both for patients and their families, health care providers, and society are substantial [10].

Due to ongoing therapeutic advancements, there is continued optimism regarding the improvement of life expectancy [4]. As reported by Turcios [3], life expectancy has seen significant increases, rising from 41.2 years in 2015 to 47.7 years by 2016. Considering the projected 70% increase in the adult CF population between 2010 and 2025 [11], it is crucial to advance not only clinical care but also psychological support [4].

When diagnosed with multiple comorbidities that require costly and complex treatments and are associated with reduced survival rates, it is understandable that both patients and their families may experience negative reactions [12]. This is particularly evident in the case of CF, a genetic condition that shapes an individual’s life, bringing not only physical challenges but also psychological burdens [12].

Living with CF can trigger challenging emotions related to uncertainty, loss of identity, and the potential loss of social support networks, including long-standing family and friends [12]. Additionally, as CF is a chronic and debilitating disease, conditions like anxiety and depression—which have been linked to more frequent hospitalizations compared to patients without these conditions [13]—can severely impact patients’ quality of life [14]. These mental health challenges can foster less optimistic beliefs about the effectiveness of medications, consequently affecting treatment adherence [13]. Poor adherence, in turn, has been associated with higher healthcare costs [10], higher rates of depression [14], and more frequent episodes of exacerbation and hospitalizations [13].

Moreover, given that CF involves a wide range of daily treatments that may need to be carried out in public spaces (e.g., taking medications), often in front of people unaware of the condition, individuals with CF may experience a fear of stigmatization [13]. This fear has been associated with reduced pulmonary function, as well as more severe symptoms of depression and anxiety, lower levels of optimism, and a poorer health-related quality of life [13]. Additionally, frequent coughing, a common symptom of CF, can have negative social consequences, such as physical distancing by those who may perceive the coughing as a symptom of a contagious illness (e.g., a cold) [15].

The importance of research involving individuals with CF is underscored by the complexity of the condition, which presents both psychological and physical challenges, as well as the progressive increase in life expectancy associated with the disease [3]. This highlights the growing need to better understand the psychological challenges related to adapting to CF [16]. However, current research remains limited. While previous reviews exist, they are either outdated [17,18,19], focus solely on depression and anxiety [14], or concentrate only on factors that promote resilience [16].

Accordingly, this systematic review synthesized and analyzed the psychosocial factors associated with adults (≥18 years) living with CF, guided by the research question: “What psychosocial aspects of CF are documented in the current literature?” By identifying the most prominent psychosocial factors, this review provides a comprehensive update of existing evidence, characterizes current research gaps, and offers clinical insights to enhance psychological support and future investigations.

## 2. Materials and Methods

This mixed-methods systematic review followed the Preferred Reporting Items for Systematic Reviews and Meta-Analyses (PRISMA) guidelines [20] to ensure a structured and transparent approach in identifying, reviewing, and including articles for the present analysis. A review protocol was retrospectively registered into the Open Science Framework (OSF) database (registry number: 10.17605/OSF.IO/HFKVB).

### 2.1. Eligibility Criteria

The following inclusion criteria were established for this mixed-methods systematic review: (1) quantitative, qualitative, or mixed-method studies that evaluate any type of psychosocial aspects associated with living with CF in adults (except anxiety and depression, examined in [14]; if studies included anxiety and depression as part of a broader psychosocial or health-related context, those studies were included); (2) peer-reviewed articles; (3) articles written in English, Portuguese, or Spanish; and (4) articles published in the last 10 years. Exclusion criteria were (1) theoretical articles or literature reviews; (2) studies focused on scale validation; and (3) articles without full-text access (after no response from the authors) or that did not provide a translation to English, Portuguese, or Spanish.

### 2.2. Search Strategy and Study Selection

The literature search for this mixed-methods systematic review was conducted in November 2024 for different databases including Scopus, ScienceDirect, Academic Search Complete, MEDLINE, Supplemental Index, Complementary Index, APA PsycInfo, SciELO, Directory of Open Access Journals via EBSCO—Research Databases, EBooks, and Discovery Service.

The search terms used were cystic fibrosis (TI) AND social support or coping or resilience or psychological distress or mental health or emotional distress or emotion or life satisfaction or psychosocial adaptation or psychological adaptation or psychological needs or psychological well-being or stigma (all fields). To exclude studies only focused on anxiety, depression, children, adolescents, caregivers, or the focus on lung transplantation, additional terms were applied: NOT depression or anxiety NOT children or adolescents or youth or child or teenager or pediatric or caregivers or transplantation (see Supplementary Materials).

This initial search yielded a total of 701 articles. After the automatic removal of 137 duplicate articles and the application of database-built filtering options, 334 articles were excluded. These exclusions were based on the following criteria: the search was limited to peer-reviewed articles in English, Portuguese, and Spanish, and to studies published within the last 10 years. These filters were automatically applied using the built-in features of the databases, ensuring that the retrieved articles met the language, publication, and peer-review criteria.

**Figure 1:**
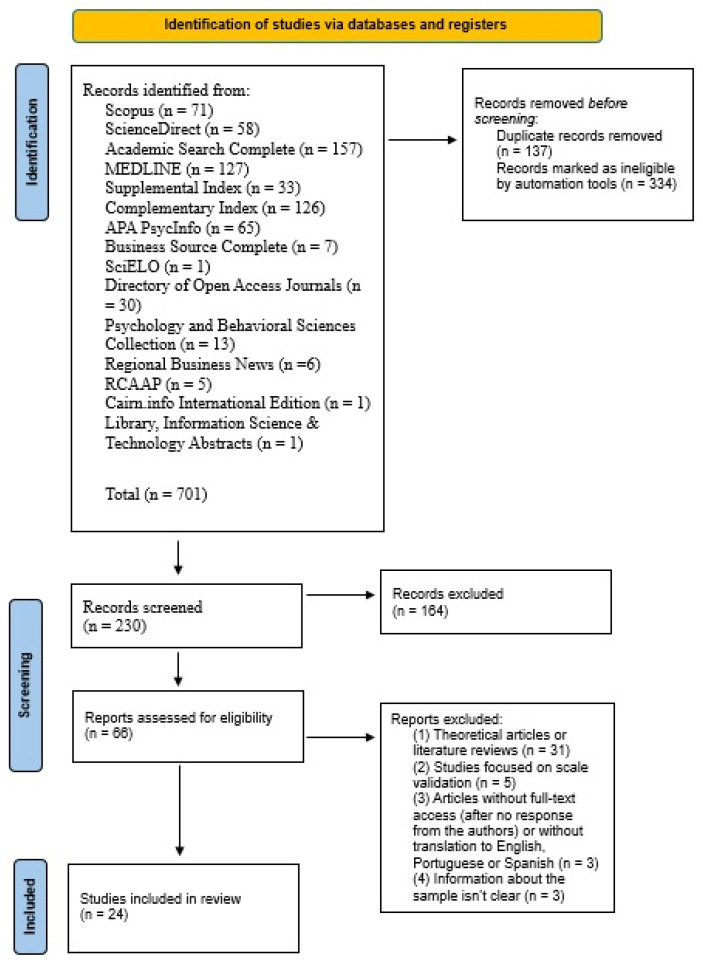
Identification and selection of studies.

Thus, a final total of 230 articles remained for further analysis. The 230 articles were screened based on their titles and abstracts, considering both the inclusion and exclusion criteria. Following this step, 66 articles remained for full-text review. During this stage, articles that did not meet the predefined inclusion criteria were excluded, resulting in the removal of 42 articles. Therefore, a total of 24 scientific articles were included in this mixed-methods systematic review (Figure 1). This was performed and independently reviewed by two researchers.

## 3. Results

The results of this mixed-methods systematic review are presented below. First, an overview of the general characteristics of the included studies is presented, including information on the authors, country, demographic characteristics of the participants, study design, types of measures used (to indicate the source of validity for each tool), and the objective of each study. Then, the data analyses and the key findings from each study are described.

### 3.1. General Description of the Articles

Table 1 presents the 24 articles included in this mixed-methods systematic review, published between 2015 and 2024. Most of the studies were conducted in the United Kingdom (n = 9) [21,22,23,24,25,26,27,28,29] and the United States of America (n = 8) [30,31,32,33,34,35,36,37]. Two studies were from Brazil [38,39], one study was from Sweden [40], one was from Australia [41], one was from Greece [42], one was from Canada [43], and one was from Israel [44].

In terms of participant characteristics, sample size ranged from a minimum of 12 participants in a cross-sectional qualitative study [39] to a maximum of 250 participants in a longitudinal quantitative study [32], yielding a total of 2023 participants across all 24 studies. Data were collected from both male and female participants in all studies, although Aguiar et al. [38] did not provide specific information regarding the sex or the age of the participants (n = 52). For the remaining 1971 participants, where sex information was available, 843 were male (42.77%) and 1128 were female (57.23%). Although there were more female participants in total, the male participants were predominant in 13 of the 24 studies included. The study of Williaws et al. [29], due to his aim, only had female participants (n = 182). The average age of the participants was 31.2 and the age range varied from a minimum of 20.7 years [23] to a maximum of 70 years [24].

Regarding the study designs of the 24 included articles, nine employed a cross-sectional quantitative design, seven used a cross-sectional qualitative design, four employed a longitudinal quantitative design, three utilized a mixed-methods cross-sectional design, and one used a mixed-methods longitudinal design. In the cross-sectional quantitative studies (n = 9) and in the longitudinal quantitative studies (n = 5), self-report scales were administered to participants. The cross-sectional qualitative studies (n = 7) used a semi-structured interview developed by the authors to address the study’s objectives. The mixed-methods cross-sectional studies (n = 3) employed both self-report scales for participants and a structured interview [23] or a semi-structured interview [28,29] to assess participants’ psychosocial functioning.

Although all the articles aimed to identify the psychosocial aspects associated with CF, they focused on different topics. These included the use of mindfulness and self-compassion [25], coping strategies [21,23,27], or hygge practices [35] to deal with the disease. Additionally, the experience of dealing with CF and overall psychological well-being [24,32,38,39,40], possible health risk behaviors [26], and treatment adherence [22,36] were also some of the themes explored. More specifically, themes like adverse childhood experiences [28], existential distress [37], and social isolation [34] within this population were studied.

In addition, there were themes like self-disclosure of the diagnosis to family, friends, romantic partners, and co-workers [30,31,44], shared decision-making of reproductive goals [29], and social support [33]. Finally, there were some studies concerning social issues, like the access to psychological services [43], financial costs related to the maintenance of the disease [42], and the use of telehealth [41].

### 3.2. Main Results

Table 2 presents the data analysis methods used in the 24 articles included in this mixed-methods systematic review. The seven qualitative studies [22,25,26,31,35,39,44] employed thematic coding to analyze the collected data. In contrast, the 14 quantitative studies utilized various statistical tests, including descriptive analyses to characterize the sample, Spearman correlations [30,34,38,42], Pearson correlations [37,42], bivariate correlations [24,32,33], *T*-tests [21,30,36,41,42], Bonferroni post hoc corrections [24,30], and Holm’s sequential Bonferroni procedure [43]. Additionally, multiple linear regression [33,36,42], multivariate regression [27,37], linear regression [17,32], logistic regression [36], Mann–Whitney [34,38], Wilcoxon [37,41], Fisher exact test [21,43], Kruskal–Wallis [34], structural equation modelling [40], Cox’s proportional hazards model [21], McNemar test and linear mixed effects models [41], and, at last, Kolmogorov-Sminorv and Anova [42] were used.

In the mixed-methods studies, one of them used thematic coding and descriptive analyses [23], one used only Pearson correlations and did not provide information on how they coded the semi-structured interview [28], and the last one used descriptive analysis and multivariable linear regression models to analyze the quantitative data, while thematic analysis using the COM-B model was used to treat the semi-structured interview [29].

## 4. Discussion

The main objective of this mixed-methods systematic review was to organize and analyze the literature on the psychosocial aspects associated with the experiences of patients over 18 years old with CF. This review aimed to contribute to the understanding of the psychological component of CF and to advance research on the involved psychosocial aspects. This need arises from the genetic and clinical advancements in treating CF, which have led to an increased life expectancy for this population [4], consequently introducing new challenges in adulthood.

Firstly, it is important to note that the number of studies (n = 24) published between 2015 and 2024 that were included in this mixed-methods systematic review marks a powerful statement regarding the importance that is now being given to the psychosocial experiences of adults with CF, a population whose life expectancy has increased in recent years [7]. This robust number contradicts the historical notion that psychological knowledge in CF is sparse, confirming that the field has shifted its focus beyond purely physical and biological concerns.

At least one psychosocial aspect associated with the experience of living with CF was identified in all 24 studies, with coping mechanisms [21,23,27,39], quality and nature of social support [26,29,30,31,32,33], and the challenges that affect the quality of life of patients [24,34,35,38,42,43] appearing as particularly dominant. Among the other mentioned psychosocial factors are the effects of physical activity towards psychological well-being [40], the experiences of telehealth [41], the challenges of entering adulthood, particularly in relation to financial matters [25], a lack of access to effective mindfulness practices tailored for CF patients [25], difficulty in implementing self-compassion [25], and challenges in connecting mind and body [25]. Furthermore, the effects of child maltreatment and household dysfunction [28], the adherence of treatment [22,23,36], the effects of symptom burden [37], and the disclosure styles and their psychosocial implications [44] were also highlighted.

Regarding the individual psychological experience, coping mechanisms appeared as a dominant theme. Optimist coping was the most used strategy [23,39] and the only coping measure that showed a beneficial effect on survival, especially in women [21], whilst active coping was positively associated with social quality of life [27]. Interestingly, the practice of hygge, a concept centered on consciously creating coziness, comfort, and connection, appeared as a valuable strategy for promoting wellness. This practice enhanced resilience and overall quality of life, allowing patients with CF to re-establish a degree of perceived control over their health condition [35].

However, the review also highlighted challenges in adjustment. While overall levels of psychological distress (anxiety and depression) were reported as low [23], depression emerged as predominant, with patients reporting they would use psychological services if available [43]. Importantly, psychological distress appears to stem less from general adverse childhood experiences and more from illness-related trauma, which was highly prevalent [28]. Specifically, distress related to painful procedures and forced treatments during childhood emerged as a more significant burden than typical household dysfunction [28]. Furthermore, although patients recognize the protective value of self-compassion for quality of life [24] and sexual satisfaction [38], they reported significant barriers in its practical application and a perceived lack of access to relevant therapeutic tools like mindfulness [25].

This complexity in adjustment is also visible in health risk behaviors. Patients reported engaging in behaviors such as smoking and drinking, which resulted from complex interactions between identity, social support, and attitudes towards CF [26]. Notably, there was a reported lack of knowledge on the consequences of these behaviors, with many patients declaring not being informed of these by clinicians [26].

The psychological dimension deeply influences treatment adherence; while participants in general felt that their levels of adherence were reasonably high [23], the ones with a higher baseline gratitude were significantly more likely to demonstrate persistent adherence [36]. Actually, poor adherence appeared to be often rooted in opportunity and capability barriers rather than purely psychological resistance; individuals with very low adherence frequently cited logistical factors (e.g., lack of time, privacy, or financial resources) as the primary challenge [22]. Conversely, social influences—specifically the comfort and willingness to perform treatments openly—act as a strong enabler for high adherence [22]. Self-efficacy regarding medication adherence was also shown to be positively correlated with undergoing treatments in front of loved ones [30].

Regarding the physical outcomes, the use of medication ensured better physical health [42], whilst a better physical condition resulted in better sexual activity [38]. These findings regarding the connection between physical action and well-being were in contrast to the ones from Bäckström-Eriksson et al. [40], where the authors noted that there seems to be no advantage in performing physical exercise concerning psychological well-being if not accompanied by a positive effect on medical status.

Navigating the social world remains a central challenge. While CF had no reported negative effect on friendships, being admitted to the hospital and managing treatments impacted work or studies [23], whilst support from loved ones and the professional team represented a crucial buffer against these difficulties [39]. Women reported more social support than men, and being employed was also associated with greater perceptions of support [33]. Greater social support was associated with less treatment burden and a better overall perception of health [32]. However, subjective social isolation—the feeling of loneliness—significantly predicted poorer quality-of-life outcomes, particularly among unmarried individuals, suggesting that the perception of loneliness leads to behavioral withdrawal [34].

A key component of social interaction is disclosure. Many patients used more than one disclosure strategy during their lifetime, depending on life transitions like marriage, having children, or disease worsening [44], while participants typically adopted the disclosure method of their parents. Nonetheless, selective disclosure was predominantly used to reduce negative consequences like pity, insults, or discrimination [44]. While patients experienced a greater comfort sharing their diagnosis with loved ones [30], shame and embarrassment due to symptoms like coughing may still lead to difficulties in maintaining romantic relationships [39].

Finally, this review highlights systemic gaps. The findings indicate that the unexpectedly prolonged life trajectory leaves CF adults unprepared for key life transitions, with financial issues being a major concern [25]. Patients reported unmet needs predominantly in the physical, daily living, and psychological domains, with concerns such as fatigue and fears about disease progression being highly prevalent [37]. Furthermore, while participants expressed a high motivation to engage in shared decision-making (SDM), their capacity was compromised by structural barriers, specifically insufficient information about reproductive goals (e.g., pre-pregnancy preparations), indicating that healthcare teams must proactively address these gaps [29]. On a positive note, telehealth delivery offered a comparable quality of care to in-person visits, while reducing costs and improving emotional functioning [41,42].

All of these interconnected psychosocial aspects and their consequences on the mental and physical health of individuals with CF highlight the importance of interventions that address both clinical and psychosocial aspects—from those most studied in this population (e.g., anxiety and depression) to those less explored (e.g., stigma, self-esteem, symptom burden).

In conclusion, this mixed-methods systematic review has fulfilled its objective: to organize and analyze the psychosocial aspects associated with living with CF in individuals over 18 years old. In doing so, it has contributed to the current understanding of what is known and the path that remains in terms of the psychosocial aspects impacting the lives of adult CF patients.

### 4.1. Limitations

Although this mixed-methods systematic review identifies and updates information on the psychosocial aspects that may be involved in the experience of living with CF and provides useful insights into the gaps in this area of study, several limitations must be acknowledged.

A limitation of this review is the absence of formal inter-rater reliability testing (e.g., kappa analysis) during the full-text screening and data extraction process. While discrepancies between reviewers were resolved through discussion and mutual agreement, the lack of formal reliability measures may limit the reproducibility and consistency of the screening process.

Also, 17 of the 24 studies were conducted predominantly in the same countries—nine in the United Kingdom and eight in the United States of America—with the remaining seven being conducted in Brazil (n = 2), Sweden (n = 1), Australia (n = 1), Greece (n = 1), Canada (n = 1), and Israel (n = 1). Therefore, most of the studies (n = 20) originate from only two continents, Europe and North America, specifically in the Northern Hemisphere. This limitation may introduce a cultural bias in the results and interpretations of this review. While this may be partially explained by the prevalence of the disease in the Caucasian population [5] and the lack of recent implementation of neonatal screening for CF diagnosis in some of the excluded continents, it limits this mixed-methods systematic review in addressing the psychosocial aspects of living with CF in non-Western cultures. Furthermore, according to the latest record from the Cystic Fibrosis Foundation, which collects data solely in the United States, more than 17% of the 33.288 patients registered in the database are not of Caucasian descent, demonstrating that the CF population has diversified in recent years [9].

The use of a variety of different scales to assess similar psychosocial constructs (e.g., quality of life, anxiety, or coping) limits the ability to directly compare results or conduct a quantitative synthesis of the findings.

To conclude, the timeframe of the 24 articles included in this mixed-methods systematic review coincides with the widespread introduction of highly effective modulator therapies (e.g., CFTR modulators). The reviewed studies did not consistently control for or distinguish between patients receiving these transformative therapies and those who were not; as for this, the psychosocial experiences reported in earlier studies may differ significantly from the current reality of patients benefiting from these new treatments, potentially affecting the generalizability of the findings to the current clinical landscape.

### 4.2. Future Research and Clinical Implications

Based on the results of this mixed-methods systematic review, all studies (n = 24) identified at least one psychosocial aspect that impacts the life of an adult with CF. While the findings of this review confirm a positive shift beyond purely physical concerns, the study of psychosocial adaptation in adulthood is still a relatively recent and evolving field. This timeline mirrors the medical history of the disease: for decades, the urgent scientific priority was aimed at genetic and biological advancements to reduce early mortality [4], given that widespread survival into adulthood only became a reality after the 1980s [7].

CF affects approximately 80,000 people worldwide [5]. With increasing life expectancy, particularly due to advances in treatments with CFTR modulators [7], individuals with this disease are experiencing new phases of adulthood (e.g., entering the workforce and starting families). In fact, the life expectancy for babies born with CF between 2019 and 2023 is now 61 years, compared to 45 years for those born between 2014 and 2018 and 33 years for those born between 1999 and 2003 [9]. This data shows that adults aged 21 to 25, born between 1999 and 2003, are now entering adulthood, which presents an opportunity to address the gaps in research on CF and psychosocial aspects in adult life with a significant sample. Therefore, investigating this topic and its implications for older ages, with accompanying clinical progress and emerging psychosocial needs, becomes increasingly relevant.

The implications should not remain limited to research, as this work could enable healthcare teams involved in the treatment and management of CF (e.g., doctors, physiotherapists, nurses, psychologists) to better understand the daily challenges faced by adult CF patients. This, in turn, could lead to the adjustment of techniques and more effective support. The need for more studies on the psychosocial impact in this population is closely linked to the need to develop effective intervention programs to improve adaptation to the disease, particularly as it relates to adulthood, given the physical limitations this disease entails.

In this context, recognizing that CF is a rare lifelong disease, group intervention programs—considering healthcare guidelines to maintain necessary distance (e.g., online group interventions) and avoid cross-contamination [13]—could prove valuable. These groups foster therapeutic factors that contribute to patient change, such as instilling hope, promoting universality, and sharing information [75]. Through such interventions, CF patients could find a safe and supportive environment to share their difficulties, fears, and concerns.

Additionally, since CF is a progressive disease, although individuals are born with it and do not experience an initial adjustment to the condition, they must adapt to their deteriorating health over the years and the possibility of early mortality. Interestingly, anxiety related to death was not explored in this mixed-methods systematic review, highlighting a significant gap in research on this population. Given the progressive nature of the disease and the reality of early mortality, further research in these areas is essential to help healthcare professionals provide effective intervention, particularly in guiding patients through adulthood and preparing for potential early death.

## 5. Conclusions

This mixed-methods systematic review synthesized and analyzed the psychosocial dimensions of living with CF in adults. The findings underscore the critical importance of addressing the psychological burden alongside physical symptoms, especially as life expectancy increases due to therapeutic advancements. Key psychosocial factors—including coping strategies, social support, stigma, and mental health—were found to profoundly influence quality of life and treatment adherence in adults with CF. Furthermore, this review identifies a significant disparity in psychological support, particularly in non-Western contexts, highlighting the urgent need for integrated healthcare models that bridge clinical and psychosocial interventions. By providing a holistic understanding of the multifaceted experiences of individuals with CF, this study offers clear directions for future research and clinical practices dedicated to enhancing person-centered care.

## Figures and Tables

**Table 1:** Characteristics of the included studies.

Authors	Country	Aim	Participants	Design	Types of Measurements
Abbott et al., 2023 [21]	UK	To evaluate the role of specific ways of coping in predicting survival in CF.	n = 116 (72 ♀; 44 ♂); M_Age_ = 25.3	Quantitative; longitudinal	Survival outcome: time in days from the date of recruitment to exit from the study. Coping: Ways of Coping scale [45].
Aguiar et al., 2017 [38]	Brazil	To evaluate the correlation between 6MWT (physical performance), quality of life, and sexual satisfaction, and the differences in data between both genders in CF adult’s patients.	n = 52 (no information about age or sex)	Quantitative; cross-sectional	Quality of life: Cystic Fibrosis Questionnaire (CFQ) [46]; sexual satisfaction: sexual satisfaction questionnaire [47].
Arden et al., 2019 [22]	UK	To identify the factors affecting nebulizer adherence using the Theoretical Domains Framework (TDF), and to compare these for participants with different levels of adherence.	n = 18 (5 ♀; 13 ♂); age range = 16–31	Qualitative; cross-sectional	Adherence: semi-structured interviews.
Askew et al., 2017 [23]	UK	To determine the current health and psychosocial status of young adults with CF, to identify the perception of the challenges they face, and to assess their needs and coping strategies.	n = 45 (18 ♀; 27 ♂); M_Age_ = 20.7	Mixed-methods; cross-sectional	Challenges of CF: structured interview; anxiety and depression: Hospital Anxiety and Depression Scale [48]; coping strategies: Ways of Coping scale [45].
Backström-Eriksson et al., 2016 [40]	Sweden	To examine the associations between genetics, medical status, physical exercise, and psychological well-being in adults with CF.	n = 68 (31 ♀; 37 ♂); M_Age_ = 32.15	Quantitative; cross-sectional	HRQoL: Swedish translation of the teen/adult version of Cystic Fibrosis Questionnaire [49]. Anxiety and depression: Hospital Anxiety and Depression Scale (HADS) [50].
Bell et al., 2024 [41]	Australia	(1) To evaluate the patient experience of multidisciplinary outpatient care through telehealth with videoconferencing compared with their own recent in-hospital face-to-face care; (2) to assess the cost and time burden of each service delivery model and patient quality of life.	n = 75 (37 ♀; 38 ♂); M_Age_ = 36	Quantitative; longitudinal	Baseline: Cystic Fibrosis Questionnaire—Revised [49]; Cystic Fibrosis Foundation’s CF patient experience of care survey (assessing face-to-face service delivery model). Six months of virtual clinics: CFQ-R. Twelve-month visit: Cystic Fibrosis Foundation’s CF patient experience of care survey (assessing virtual clinic service delivery model), purpose-built cost and time commitment questionnaire comparing in-hospital face-to-face and virtual clinics from a patient perspective, CFQ-R, Telehealth Satisfaction Scale, and general feedback regarding patient model of care preference.
Borschuk et al., 2016 [30]	USA	To quantify CF disclosure (i.e., to romantic partners, co-workers, work bosses, or friends) and examine associations between disclosure and psychosocial and health outcomes (i.e., social and emotional functioning, social support, depressive symptoms, lung functioning, medication adherence and self-efficacy, and body mass index).	n = 128 (60 ♀; 68 ♂); M_Age_ = 29	Quantitative; cross-sectional	Disclosure: Cystic Fibrosis Disclosure Questionnaire (CFDS) [51,52]. Social support: Medical Outcomes Study Social Support Survey (MOS-SS) [53]; emotional and social functioning: 5-item Emotional Functioning and 6-item Social Functioning subscales of the 50-item CFQ—Revised [49]; depression: The Center for Epidemiological Studies—Depression (CES-D) [54]; self-efficacy: The Medication Self-Efficacy Questionnaire—General Subscale [55]; medication adherence: data from each participant’s local pharmacies using a compound medication possession rate (CMPR) calculation.
Broekema & Weber, 2017 [31]	USA	In what ways do individuals with CF manage the privacy concerns they have about their CF-related information when sharing this information with a romantic partner?	n = 13 (10 ♀; 3 ♂); age range: 24–43	Qualitative; cross-sectional	CF-related information and psychosocial aspects: semi-structured, open-ended interview.
Cordeiro et al., 2018 [39]	Brazil	To understand the experience of adults living with CF.	n = 12 (6 ♀; 6 ♂); M_Age_ = 26	Qualitative; cross-sectional	CF-related information and psychosocial aspects: semi-structured interviews.
Dainavas et al., 2024 [42]	Greece	To analyze the financial consequences of having CF on Greek patients, evaluate their general state of health, and specifically investigate the impact of living expenses on their quality of life.	n = 105 (49 ♀, 56 ♂); M_Age_: 32.1)	Quantitative; cross-sectional	Quality of life: Short-Form Questionnaire-36 (SF-36) [56] and CFQoL [57].
Flewelling et al., 2019 [33]	USA	Examine factors associated with social support in adults with CF to identify those who are most likely to experience a lack of support.	n = 233 (140 ♀, 93 ♂); M_Age_: 33.63)	Quantitative; longitudinal	Social support: Interpersonal Support Evaluation List (ISEL) [58].
Flewelling et al., 2019 [32]	USA	To explore the relationships between social support, mental health, physical health, treatment activity, and disease-specific quality of life in a sample of adults with CF.	n = 250 (153 ♀, 97 ♂); M_Age_: 34.67)	Quantitative; longitudinal	Social Support: Interpersonal Support Evaluation List (ISEL) [58]. Mental and physical health symptoms: Memorial Symptom Assessment Scale (MSAS) [59]; treatment activity: daily frequency of airway clearance treatments, Tool for Adherence Behavior Screening (TABS; subscale of Beliefs and Behavior Questionnaire, BBQ) [60], Health-Related Quality of Life (HRQoL); CFQ-R.
Gulledge et al., 2023 [34]	USA	To gain a preliminary description of if, and how, the CF population experiences objective and subjective social isolation.	n = 34 (25 ♀, 9 ♂); M_Age_: 35.3)	Quantitative; cross-sectional	Subjective social isolation: Patient-Reported Outcomes Measurement Information System (PROMIS) Social Isolation Short Form. Objective social isolation: Lubben Social Network Scale-Revised (LSNS-R).
Kauser et al., 2022 [25]	UK	(1) To explore the current psychosocial challenges faced in adulthood life by this relatively new adult CF population; (2) to understand and explore attitudes and experiences of mindfulness and self-compassion.	n = 20 (11 ♀; 9 ♂); M_age_ = 37	Qualitative; cross-sectional	Psychosocial challenges: semi-structured interview.
Kauser et al., 2022 [24]	UK	(1) To explore the relationships between quality of life, negative emotional states (depression, anxiety, and stress), self-compassion, and self-criticism; (2) to explore the moderating effects of self-compassion and self-criticism on the relationship between quality of life and negative emotional states within an adult CF population.	n = 114 (56 ♀, 58 ♂); M_age_ = 32.36	Quantitative; cross-sectional	Self-compassion: Self-Compassion Scale (SCS) [61]; self-criticism: The Functions of Self-Criticizing/Attacking Scale (FSCS) [62]; depression, anxiety, and stress: The Depression Anxiety Stress Scale (DASS) [63]; quality of life: CFQoL [64].
Keyte et al., 2020 [26]	UK	To explore beliefs associated with health risk behaviors within an adult CF.	n = 24 (8 ♀, 16 ♂); M_age_ = 34	Qualitative; cross-sectional	Psychosocial challenges focused on health risk behaviors: semi-structured interview.
Mc Hugh et al., 2016 [27]	UK	Examine which specific coping styles were positively or negatively associated with social and emotional QOL in a CF sample.	n = 122 (86 ♀, 36 ♂); M_age_ = 28.9	Quantitative; cross-sectional	QoL: CF Questionnaire-Revised (CFQ-R) [65]; coping styles: Brief COPE [66].
O’Leary et al., 2022 [28]	UK	To examine the prevalence of adverse childhood experiences (ACEs) in adults with CF.	n = 80 (29 ♀, 51 ♂); M_age_ = 32	Mixed-methods; cross-sectional	ACEs: Adapted version of the Centers for Disease Control and Prevention (CDC) short-form ACE questionnaire; depression: Patient Health Questionnaire (PHQ-9) [67]; anxiety: GAD-7 [68]; psychological well-being: semi-structured psychosocial interview.
Pakhale et al., 2015 [43]	Canada	(1) The extent to which adults with CF access psychological services as part of their CF treatment and care; (2) the likelihood of accessing psychological services if they were available at the CF clinic, and the concerns considered for discussion with a psychologist; (3) the likelihood that adults screened positively for depression or anxiety would utilize psychological services if they were available at the clinic.	n = 45 (19 ♀, 26 ♂); M_age_ = 30.7	Quantitative; cross-sectional	Depression: Center for Epidemiologic Studies Depression Scale (CES-D); anxiety: Generalized Anxiety Disorder (GAD-7); psychological needs: short form with three psychological needs assessments.
Pitts et al., 2024 [35]	USA	To explore how adults with CF use hygge practices to promote wellness and cope with their progressive disease.	n = 15 (13 ♀, 2 ♂); M_age_ = 36	Qualitative; cross-sectional	Psychosocial aspects: semi-structured telephone interviews.
Sherman et al., 2025 [36]	USA	To evaluate relationships between trait gratitude and long-term self-reported adherence to ACT (airway clearance therapy) among adults with CF.	n = 66 (30 ♀, 36 ♂); M_age_ = 27.15	Quantitative; longitudinal	Trait gratitude: Six-item Gratitude Questionnaire-6 (GQ-6) [69]; persistent adherence to ACT: Cystic Fibrosis Treatment Questionnaire (CFTQ) [70]; emotional well-being: CFQ-R [65].
Trandel et al., 2019 [37]	USA	(1) To determine the prevalence of existential distress in individuals with CF; (2) to investigate potential associations between symptom burden and unmet needs in this population.	n = 164 (72 ♀, 92 ♂); M_age_ = 29	Quantitative; cross-sectional	Existential distress: five items from the Supportive Care Needs Survey Short Form 34 (SCNS-34) [71]; symptom burden: The Edmonton Symptom Assessment Scale (ESAS) [72].
Werner et al., 2019 [44]	Israel	To examine the use of different disclosure strategies by adults with CF, and the psychosocial implications of these strategies.	n = 42 (16 ♀, 26 ♂); M_age_ = 31.7	Qualitative; cross-sectional	Psychosocial aspects focused on living and coping with CF and disclosure styles: semi-structured interviews.
Williams et al., 2023 [29]	UK	To investigate how capability, opportunity, and motivation can influence women’s participation in SDM (shared decision-making) relating to their reproductive goals.	n = 182; M_age_ = 31.9; online survey n = 21; age range: 26–45; qualitative interview	Mixed-methods; cross-sectional	Disease-related quality of life: six items of the CFQ-R [49]; reproductive goals, SDM behavior, SDM capability: a mix of themed questions; SDM opportunity (social environment/social support): measurement from EN-RICHED study; SDM motivation (SDM attitudes: one item of the Control of Preferences Scale [73] and decision self-efficacy: seven items of Decision Self-efficacy Scale [74]; experiences of women with CF of SDM: semi-structured interviews.

Note: Other measurement instruments were used, namely to evaluate the physical health of participants (i.e., FEV1%, FVC, and BMI) and to evaluate psychosocial aspects of other populations under study (i.e., caregivers of patients with CF); however, only those who evaluated psychosocial aspects of patients with CF are presented in this table, meeting the objective of this mixed-methods systematic review. Additionally, instruments used to measure variables related to the COVID-19 pandemic were removed. Please note that citations provided in the “Types of Measurements” column refer to the original validation or primary reference sources for each assessment instrument.

**Table 2:** Main results found in the included studies.

Authors	Data Analysis	Main Results
Abbott et al., 2023 [21]	Descriptive statistics; *T*-test; Fisher’s exact test; Cox’s proportional hazards model.	Hopefulness, distraction, or avoidance coping strategies were not associated with survival. Only optimistic coping influenced survival, even after adjusting for age, sex, and the remaining coping variables. Men with above-average levels of optimism demonstrated noticeably better survival compared to those with below-average optimism, a pattern that was also observed among women. However, the survival difference between higher and lower optimism levels was more pronounced in women, with women reporting low optimism exhibiting the poorest survival outcomes overall.
Aguiar et al., 2017 [38]	Descriptive statistics; Spearman’s correlation; Mann–Whitney.	CFQ domains 1 (physical role), 3 (vitality), 6 (body image), 9 (health perceptions), and 11 (respiratory symptoms) and sexual satisfaction questionnaire showed a positive correlation for answers about sex drive, self-confidence, willingness to initiate sexual activity, foreplay, and ability to reach orgasm. Male participants had better scores in the emotional CFQ domain, better performance, and a higher score in SSQ as well as physical performance. A correlation between sexual satisfaction and quality of life was shown, and the best scores in QOL were linked to better scores in SS. There was a relationship between physical performance and sexual satisfaction, where a better physical condition resulted in better sexual activity.
Arden et al., 2019 [22]	Framework analysis.	A total of ten factors were identified as varying according to adherence levels. Skills, memory, and decision-making and behavioral regulation were categorized under capability; environmental context and resources and social influences were classified under opportunity; and beliefs about consequences, beliefs about capability, reinforcement, social role, and identity, intentions, optimism, and emotions were grouped within motivation. Regarding factors associated with opportunity, environmental context and resources were primarily reported among individuals with very low adherence, whereas social influences were more frequently observed in individuals with a very low to low adherence. An exception was the willingness to take treatment in the presence of others, which was predominantly associated with a high adherence. With respect to motivational factors, individuals with moderate adherence appeared to be the most affected.
Askew et al., 2017 [23]	Descriptive statistics; thematic coding.	Most of the participants felt that they were knowledgeable about their medications, nutrition, and physiotherapy and felt that they were “very or extremely independent” in taking treatments. Self-reported adherence was reasonably high. Levels of psychological distress (anxiety and depression) were low. The predominant psychological coping strategy employed was optimistic acceptance. Overall, most of participants said that CF had no effect on their social or family lives or friendships. However, being admitted to the hospital, receiving treatments, and having symptoms had a negative impact on work or studies.
Backström-Eriksson et al., 2016 [40]	Descriptive statistics; structural equation modelling (SEM). maximum likelihood with robust SE estimation.	There is a psychological disadvantage for having the mutation classes, since it causes a more severe disease increase with age. Furthermore, a large portion of this increase in disadvantage is accounted for by a matching deterioration in medical status. It may be that there is no advantage in performing physical exercise when considering psychological well-being if not combined with a positive effect on medical status.
Bell et al., 2024 [41]	Descriptive analysis; *t*-test or Wilcoxon signed rank test; unpaired *t*-tests or linear mixed-effects models; McNemar test.	Experience of care (in-person or telehealth) and QoL showed no difference. Time and cost related to travel to the clinic and sometimes accommodation was reduced for some of the participants. Most of the participants felt comfortable using telehealth with videoconferencing and felt that their privacy was respected during these encounters.
Borschuk et al., 2016 [30]	Descriptive statistics; Spearman’s correlations; *T*-tests; Bonferroni post hoc corrections.	Participants reported a high level of comfort disclosing their diagnosis, comfort undergoing treatments in front of their romantic partners, and only some comfort undergoing treatments in front of close friends. In turn, social support, as well as emotional and social functioning, were not related to disclosing the diagnosis, but to comfort in talking about the disease and undergoing treatments in front of others. Depressive symptoms were unrelated to disclosing the diagnosis and only slightly associated with comfort in undergoing treatments in front of romantic partners, demonstrating that lower levels of depressive symptoms were associated with greater comfort. Self-efficacy regarding medication adherence was shown to be positively correlated with undergoing treatments in front of loved ones. Finally, this study demonstrated that participants working with the disease in a more advanced stage with more severe lung function, and, in turn, with more visible physical symptoms and more recurrent hospitalizations, were more likely to reveal the diagnosis to their bosses than those in a more stable state of health. In turn, few participants revealed the diagnosis to their coworkers.
Broekema & Weber, 2017 [31]	Grounded theory.	While making the decision to disclose the disease, participants highlighted that they weigh the risks (lack of trust and fear of rejection) and the benefits (feeling accepted by a partner and being honest with oneself and with a partner). One of the reasons for the disclosure of CF and any related information was the unpredictable nature of the disease (triggered rule), but normally, when the health status became more severe, the participants with CF would reveal more information with their romantic partner in a shorter amount of time. Furthermore, the participants emphasized that the need for social support and stability in a romantic relationship superseded other needs and desires, such as happiness and compatibility. Finally, experiences related to past disclosure have led some participants to withhold information from romantic partners and people in general. Mostly, outsiders had negative reactions to the CF-related information, which led the person with CF to develop rules to self-protect in future disclosures. In some cases, the privacy of what was told to the romantic partner suffered some turbulence, since they told others outside the relationship (e.g., parents), and the reactions included teasing and parental concern.
Cordeiro et al., 2018 [39]	Social phenomenology.	The following categories revealing the context of meanings attributed by adults with CF in daily living to the disease were evidenced: (1) the biopsychosocial impact of the disease on daily life: some of the referenced challenges included fatigue, the inability to perform activities of daily living, and the need to use multiple medications and treatment time demands, leading to school or work dropout and social isolation; (2) social prejudice as a source of embarrassment: due to frequent cough, people associate CF with contagious and serious diseases, which causes shame and embarrassment for adults living with it; additionally, it may lead to difficulties in maintaining a romantic relationship; (3) coping strategies: most participants reported that they are positive, optimistic, resilient, and are always searching for strategies to overcome the limitations imposed by the disease; furthermore, the support of loved ones and the professional team represents a support to face the difficulties experienced; (4) fear, uncertainties, and the desire to carry out life projects: most of the participants reported that the wait to receive a transplant represented a difficult moment for them, leading to uncertainties about their own life, the surgery, and if they were going to make it, while also being concerned about their loved ones. Nonetheless, participants expected that, after lung transplantation, they would have autonomy and independence, while also being able to get married, build a family, have children, find a good job, resume their studies, and perform the activities that give them pleasure.
Dainavas et al., 2024 [42]	Descriptive statistics; Kolmogorov–Smirnov test; T-student test; ANOVA; Pearson’s correlation; Spearman’s correlation; multiple linear regression model.	Reducing overall costs due to the disease improved the emotional functioning of the patients; also, they had fewer apprehensions regarding the future and had a better social life and a superior treatment quality of life. Male employees had better physical health, whereas patients (male or female) receiving the medication also had better physical health. Individuals with a greater monthly household income, male participants, and those receiving pharmacological treatment demonstrated better functional capacity and physical performance. Overall, the study population reported quality of life levels below the normative average, affecting both mental and physical health domains.
Flewelling et al., 2019 [33]	Descriptive statistics; bivariate correlations; multiple linear regression model.	Participants that were females reported more social support than male participants. Being employed was also associated with greater perceptions of social support. In contrast, age, income, education, marital status, and disease severity were not associated with perceptions of social support.
Flewelling et al., 2019 [32]	Descriptive statistics; unadjusted bivariate correlations; adjusted linear regression.	Greater social support was associated with less treatment burden and better overall perceptions of their health, fewer self-reported mental and physical health symptoms, digestive symptoms, and eating disturbances over time, elevated emotional, social, and role functioning, as well as vitality and improved body image. In contrast, social support did not predict physical functioning, problems gaining weight, respiratory symptoms, or higher treatment activity.
Gulledge et al., 2023 [34]	Descriptive statistics; normality tests; Spearman’s correlation; Mann–Whitney; Kruskal–Wallis.	A statistically significant negative correlation was observed between subjective isolation scores and objective isolation scores, indicating that participants with higher levels of subjective social isolation (SOI) engaged in less social interaction. Subjective SOI scores were significantly higher among individuals who were unmarried or not living with a partner. Objective SOI scores were significantly higher among women. Additionally, a significant negative correlation was identified between subjective SOI scores and the physical, emotional, treatment burden, health, social, body, and role domains of the CFQ-R, indicating that a greater subjective SOI was associated with poorer quality-of-life outcomes. In contrast, objective SOI scores were positively correlated with the CFQ-R emotional domain, suggesting that larger social networks were associated with better emotional quality of life.
Kauser et al., 2022 [25]	Thematic analysis.	Participants in this study demonstrated that they were unprepared for adult life (especially about financial issues) and revealed concerns about it, despite feeling grateful for exceeding the average life expectancy of patients with CF. The results of this study also revealed the reduced capacity of these participants to connect mind and body in understanding their illness, as well as problems in the areas of body awareness due to weight disproportion and its impact on the identity of each of these participants. Additionally, participants reported a lack of access to information related to mindfulness practice for patients with CF. Finally, participants recognized the importance of self-compassion in living with the disease and treatment; however, the difficulty was related to implementing it in their own lives.
Kauser et al., 2022 [24]	Descriptive statistics; normality and linearity; bivariate correlations; moderation analyses; Bonferroni correction procedure.	In general, participants who showed a better quality of life reported greater self-compassion, whereas the ones who demonstrated more severe negative emotional states showed greater self-criticism. As for the moderator effects, participants that felt more positive about their body image demonstrated lower levels of anxiety when self-compassion moderated the relationship. Also, people that showed fewer treatment-related issues reported lower levels of anxiety when self-compassion moderated the relationship. Finally, participants that had more positive career experiences reported lower levels of stress when self-criticism moderated the relationship.
Keyte et al., 2020 [26]	Thematic analysis.	Five themes around health risk behaviors (1): “All I’ve ever wanted is to be happy”—participants showed that smoking and drinking helped them with the difficult aspects of the disease, since it allowed them to escape and seek pleasure (life-oriented illness perspective) just for fun or enjoyment; (2) “Are they sure I haven’t just got asthma?”—some patients reported that, since their adverse health effects were absent, there was no need to be careful, so the avoidance of CF played an important role while engaging in health risk behaviors; (3) “If you haven’t got support it can be detrimental to you because you need that support”—there were moments when the participants were influenced to make health risk behaviors by their peers and they felt the need to do it, since they need social support; some of them said that, if they had social support that did not promote these behaviors, it would be easier; (4) “Why should I let CF completely dominate my life?”—the participants showed a desire for normalcy, and they valued living their life in a way that was fulfilling, even if it included health risks behaviors; (5) “The biggest challenge is coming to terms with having CF”—some participants stated that it was when they were more depressed that they engaged in health risk behaviors, while others said that they did these behaviors because they did not experience any adverse health effects. These health risk behaviors helped individuals maintain consistency with their group identity while reducing stress, thereby supporting psychological well-being. Overall, health risk behaviors are the result of complex interactions between identity, social support, challenges, acceptance, and attitudes towards CF. Furthermore, there was a lack of knowledge on the consequences of these behaviors, with many participants reporting not being informed of these by clinicians.
Mc Hugh et al., 2016 [27]	Descriptive statistics; multivariate regression model.	Distraction coping demonstrated a negative association both with emotional and social quality of life. Additionally, higher substance use and disengagement were associated with lower emotional quality of life. In contrast, greater religious coping, instrumental support, and acceptance were positively associated with emotional quality of life. Finally, active coping was positively associated with social quality of life.
O’Leary et al., 2022 [28]	Pearson’s correlation.	Regarding the overall prevalence of ACEs (not including exposure to medical/health-related trauma) most of the sample experienced 0 to 1 ACEs, with only a few reporting 2–3 or 4 ACEs. The categories that had more prevalence were child maltreatment, especially verbal abuse, household dysfunction, and parental separation. Finally, illness related trauma in childhood was prevalent within more than half of participants, with reports of a painful or frightening medical procedure and the feeling of being forced to have treatment or a procedure.
Pakhale et al., 2015 [43]	Descriptive statistics; Fisher exact tests; Holm’s sequential Bonferroni procedure.	Between anxiety and depression, the latter was the more significant of the two in this sample, with only a few of the participants reporting having them both. Past access to psychological services in CF care was not significantly related to participants’ levels of depressive symptoms or anxiety. Most of the patients reporting elevated symptoms of depression and/or anxiety indicated they would be likely to use psychological services if available at the clinic. The majority of those with low depression symptoms reported that it was unlikely they would use psychological services, but almost half of those reporting low anxiety reported that they would be likely to access those services if available. The concerns considered most relevant by the sample to discuss with a psychologist were worries, mood, life stress, adjustment to CF, life transitions, and quality of life.
Pitts et al., 2024 [35]	Descriptive phenomenology; Colaizzi’s phenomenological method.	The majority of the sample reported having anxiety or depression related to their CF diagnosis. There were three major themes, each with three subthemes: (1) aesthetics—cozy environment, comfort and peace, candles; (2) attitudes—emotional benefits, mindfulness, holistic health; (3) activities—nature, including others, feasibility. In general, hygge practices deeply impacted the physical and emotional experience of living with CF, since practicing hygge fosters a sense of peace, comfort, and tranquility, cultivates resilience, and promotes coping for individuals facing a chronic illness with a high treatment burden. In this study, participants described hygge as enhancing their overall quality of life, well-being, and perceived control over CF.
Sherman et al., 2025 [36]	Descriptive statistics; *T*-tests or chi-square; logistic regression; multiple regression.	Nearly half of the participants demonstrated persistent adherence to airway clearance therapy (ACT), whereas the remaining reported poor persistence. Participants who reported higher levels of gratitude at baseline were significantly more likely to maintain consistent adherence throughout the one-year period. Gratitude levels showed substantial variability among individuals and continued to be a significant predictor of ACT adherence, even after controlling for other established psychosocial resources, including social support and emotional well-being.
Trandel et al., 2019 [37]	Descriptive statistics; Pearson’s correlations; Wilcoxon rank-sum test; multivariable regression model.	The majority of patients reported experiencing at least a mild symptom burden related to the disease. Similarly, most respondents indicated at least one unmet palliative care need. The three most prevalent unmet needs were “lack of energy/tiredness,” “feeling unwell most of the time,” and “fears about CF worsening.” The first unmet need outside the physical and psychological domains ranked fifteenth in prevalence. Symptom burden was significantly correlated with all SCNS-34 domain scores, with particularly strong correlations observed for the physical, daily living, and psychological domains. In the subsample of participants reporting moderate-to-severe symptoms, depressive symptoms were significantly associated with higher scores across all five SCNS-34 domains, while anxiety symptoms were associated with higher scores in four domains, with the exception of sexuality.
Werner et al., 2019 [44]	Content analysis. Two methodologies: (1) Partially focused analysis, based on Corrigan and Lundin’s model; (2) participant-focused analyses, following and inductive and open-coding process.	Two main themes related to CF disclosure: (1) disclosure styles and their psychosocial implications, and (2) perceptions of the differences between previous and current disclosure styles. There were some patients that described using more than one disclosure strategy at different stages of their lives. Nonetheless, social avoidance was the least mentioned style and was only used in childhood, while the most common style was selective disclosure. Some participants associate the act of revealing with feelings of shame and negative effects on romantic relationships. Also, secrecy allowed people with CF to remain in control of who knew about their illness, minimizing negative outcomes such as pity, insults, and discrimination. Overall, participants characterized their disclosure styles in relation to patterns established by their parents during childhood or to modifications they introduced later in life as a result of major life events such as marriage, parenthood, or declining health.
Williams et al., 2023 [29]	Descriptive analysis; multivariable linear regression models. Thematic analysis.	Regarding this sample, women who already had children had a higher decision self-efficacy, better perceived health, and rated the extent to which their preferences for having children had been considered by their healthcare team more highly than those who did not have children. There were no differences regarding decision control preferences. Generally, learning more information about the preparation for a pregnancy (e.g., changing medications) was a topic that registered a higher importance. Women reporting more social support, who had been college educated, and were older had higher levels of decision self-efficacy, highlighting inequalities. Women with higher decision self-efficacy reported better experiences of shared decision-making related to their reproductive goals. Finally, interviews indicated that women were highly motivated to engage in shared decision-making, but their capability was compromised by a lack of information and the perception of insufficient opportunities for focused discussions about shared decision-making.

## Data Availability

No new data were created or analyzed in this study. Data sharing is not applicable to this article.

## Supplementary Materials

The following supporting information can be downloaded at: https://www.mdpi.com/article/10.3390/healthcare14030351/s1, PRISMA 2020 Checklist.
